# Identifying common genetic etiologies between iridocyclitis and related immune-mediated diseases

**DOI:** 10.3389/fimmu.2025.1755047

**Published:** 2026-01-15

**Authors:** Zhao Liu, Wenqiao Zhang, Qiuyuan Wang, Xumei Du, Zhiqi Tao, Jianguo Huang, Yuqin Wang

**Affiliations:** 1 National Clinical Research Center for Ocular Diseases, Eye Hospital, Wenzhou Medical University, Wenzhou, China; 2 State Key Laboratory of Ophthalmology, Optometry and Visual Science, Eye Hospital, Wenzhou Medical University, Wenzhou, China; 3 Special Key Laboratory of Ocular Diseases of Guizhou Province, Zunyi Medical University, Zunyi, China

**Keywords:** autoimmune uveitis, genetics, GWAS, IL15RA, immune-mediated diseases, meta, FBXL18

## Abstract

**Background:**

Patients with iridocyclitis are at heightened risk for immune-mediated diseases. The genetic underpinnings of iridocyclitis are intricate, necessitating an integrated approach to unravel the genetic connections between iridocyclitis and these diseases.

**Methods:**

GWAS data were integrated from three databases using METAL. Independent risk loci were analyzed through conditional and joint genome-wide multi-trait analysis, multi-marker genomic annotation, and functional mapping of significant loci. This approach combined quantitative trait loci data and various methodologies to identify genes and proteins associated with risk. Target gene verification was conducted through cell experiments and flow cytometry.

**Results:**

The study identified five independent iridocyclitis-related risk loci and 123 associated genes. Additionally, 14 multi-disease risk genes and 109 disease-related proteins were discovered. Flow cytometry confirms that FBXL18 and IL15RA are responsive to inflammatory stimuli and supports their role in immune-mediated pathways relevant to iridocyclitis, underscoring its potential as a therapeutic target.

**Conclusion:**

This study indicates that the polygenic factors shared between iridocyclitis and immune-mediated diseases are broadly distributed across the genome. These findings affirm a genetic link between iridocyclitis and immune-mediated diseases and highlight new therapeutic targets for these conditions.

## 1 Introduction

Autoimmune uveitis (AU) is a multifaceted and potentially severe ocular condition characterized by inflammation within the uvea and progressively leads to a spectrum of complications, including cataracts, glaucoma, macular edema, and irreversible vision loss in severe cases; however, its pathogenesis remains unclear (1). AU predominantly affects individuals in the middle-aged population, with reported rates of vision impairment ranging from 5% to 25% (2). Increasing evidence has underscored the etiological relationship between AU and systemic autoimmune disorders (3). Autoimmune anterior uveitis (AAU) accounts for most uveitis cases according to global surveys (4). Non-infectious iridocyclitis, as a type of AAU, is often linked to seronegative spondyloarthropathies, such as ankylosing spondylitis (AS), reactive arthritis, psoriatic arthritis, and inflammatory bowel diseases, including Crohn’s disease (CD) and ulcerative colitis (UC) (5). Shared genetic foundations have been identified across immune-mediated diseases, implicating polygenic susceptibility genes (6). Thus, investigations on the genetic links between Non-infectious iridocyclitis and related inflammatory conditions hold significant promises for advancing novel therapeutic interventions for iridocyclitis.

Although substantial progress has been achieved in understanding these relationships, studies concentrating on single diseases can miss crucial genetic loci and regulatory mechanisms. Thus, multitrait analysis is essential for broadening the phenotypic spectrum, pinpointing risk loci, and exploring shared genetic causes between diseases. Common genetic causes may suggest pleiotropy as a genetic confounding factor among traits (7). Cross-trait analysis using genome-wide association study (GWAS) data can identify pleiotropic genes or loci across multiple traits (8). These pleiotropic loci could serve as valuable targets for simultaneously preventing or treating multiple diseases.

**Figure 1:**
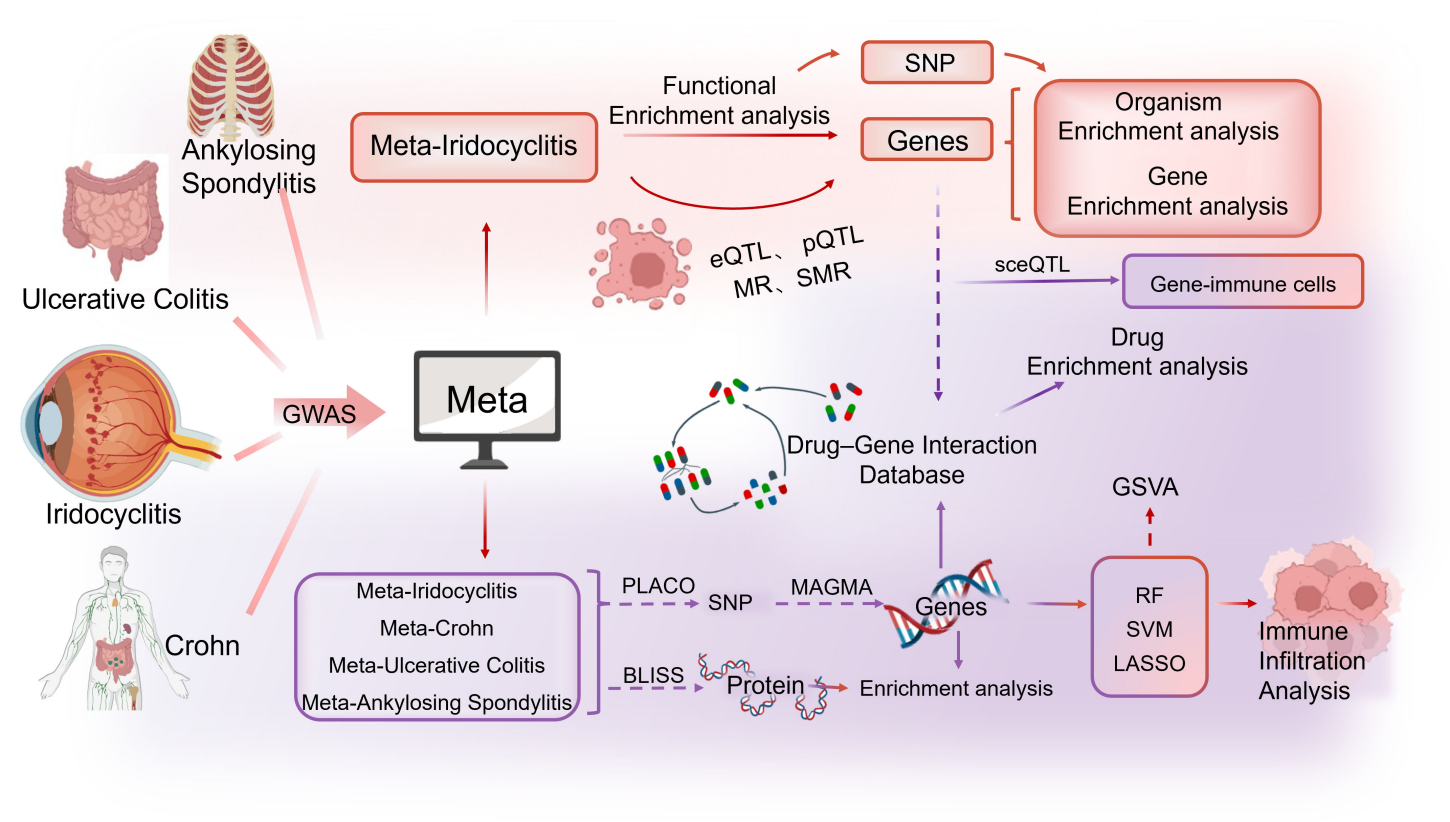
The overall study design. BLISS, Biomarker Imputation from Summary Statistics; eQTLs, Expression Quantitative Trait Loci; pQTLs, Plasma Protein QTLs; MR, Mendelian Randomization; SMR, Summary-based Mendelian randomization; SNP, Single Nucleotide Polymorphism; PLACO, Pleiotropic Analysis under the Composite Null Hypothesis; MAGMA, Multiple Marker Analysis of Genome Annotation; GSVA, Gene Set Variation Analysis; RF, Random Forest; SVM, Support Vector Machine; sceQTL, Single Cell eQTL.

This study utilized pooled data from large-scale GWASs across multiple databases (FinnGen, IEU OpenGWAS, and GWAS Catalog). Various statistical methods, including pleiotropic analysis under the composite null hypothesis (PLACO), multiple marker analysis of genome annotation (MAGMA), Mendelian randomization (MR), Summary-based Mendelian randomization (SMR), machine learning (ML), biomarker imputation from summary statistics (BLISS), and enrichment analysis, were employed to investigate pleiotropic associations between non-infectious iridocyclitis and immune-mediated diseases. Shared genetic causes were identified by focusing on single nucleotide variations, gene and protein levels, and biological pathways. In addition, genome variation analysis (GSVA) was conducted using transcriptome profiles from patients with iridocyclitis to develop a pleiotropic gene score and examine its relationship with immune cell infiltration. Drug enrichment analysis based on drug–gene interactions was performed to identify potential therapeutic agents. This study aimed to enhance current understanding of the genetic architecture of non-infectious iridocyclitis and its potential therapeutic mechanisms. **Figure 1** depicts the comprehensive design of this research.

## 2 Materials and methods

### 2.1 Data sources

GWAS summary statistics were obtained from publicly accessible datasets of individuals with European ancestry. The conditions chosen for analysis were non-infectious iridocyclitis, AS, UC, and CD. All of the data were sourced from the FinnGen database, IEU database, and GWAS catalog. The liftOver tool was employed to harmonize the data with the GRCh37 reference sequence, ensuring uniformity with the data originally aligned with the GRCh38 reference sequence (9). For each trait, we only downloaded and retained ancestry-specific European-only (EUR) GWAS releases when available (as annotated in FinnGen, IEU OpenGWAS, and GWAS catalog metadata and/or the original study documentation) and excluded multi-ancestry or transethnic GWAS summary statistics. In the contributing GWAS resources, iridocyclitis was generally defined by using diagnosis-based phenotyping (registry/electronic health record codes) and thus may aggregate multiple clinical subtypes. We therefore treated iridocyclitis as a broad noninfectious phenotype. All the information about the datasets is presented in **Table 1** and **Supplementary S1**.

**Table 1:** Data sources and sample characteristics.

Disease	Database	ID	Ancestry	Ncase	Ncontrol
Iridocyclitis	Finngen	finngen_R11_H7_IRIDOCYCLITIS	European	8957	429396
	Gwas-Catalog	GCST90043791	European	134	456214
	Gwas-Catalog	GCST90079884	European	1094	385475
Ankylosing spondylitis	Finngen	finngen_R11_M13_ANKYLOSPON	European	3484	322314
	IEU	ukb-b-18194	European	1296	461637
	Gwas-Catalog	GCST90014438	European	1344	324074
Ulcerative Colitis	Finngen	finngen_R11_ULCEROTH	European	2049	432380
	IEU	ukb-b-19386	European	1987	461023
	Gwas-Catalog	GCST90476067	European	5229	442102
Crohn’s disease	Finngen	finngen_R11_CHRONOTH	European	2553	432380
	IEU	ukb-a-552	European	732	336467
	Gwas-Catalog	GCST90475318	European	2256	313412

Quantitative trait loci (QTL) data were collected to investigate genetic links to iridocyclitis. Blood expression QTL (eQTL) information was sourced from the extensive eQTLGen consortium database, which documented the single nucleotide polymorphisms (SNPs) linked to diverse traits in 31,684 individuals (10). In addition, plasma protein QTL (pQTL) data were extracted from the deCODE database (11).

### 2.2 Meta-analysis of GWASs

This study employed METAL-Meta Analysis Helper (version: 2011-03-25) to conduct a meta-analysis of the GWAS summary statistics from each cohort (12). The overall combination was accomplished by using a sample size–weighted method.

We applied linkage disequilibrium score regression (LDSC) and reported the mean chi-squared statistic, genomic inflation factor (λGC), and LDSC intercept (with standard error) to evaluate residual population stratification and genomic inflation in the meta-analyzed GWAS summary statistics (13). We performed bivariate LDSC and reported the cross-trait intercept (with standard error) to assess potential sample overlap and/or correlated confounding between META-iridocyclitis and each immune-mediated disease in cross-trait analyses (including PLACO-based pleiotropy analyses) (14).

### 2.3 Identification of independent risk loci

The functional mapping and annotation (FUMA) platform (15) was utilized for annotation. Stringent criteria were employed, such as a maximum *P*-value of < 5 × 10^−8^ for lead single nucleotide variants (SNVs) and a broad significance threshold of *P* < 0.05. Independent SNV identification relied on an r^2^ threshold below 0.6, with lead SNVs necessitating an r^2^ below 0.1 within a 1 Mb radius. Genomic risk location was delineated by combining regions with lead SNVs less than 250 kb apart. The variants were annotated utilizing ANNOVAR (15) and assessed for potential deleterious effects based on their combined annotation-dependent depletion (CADD) scores, where values above 12.37 indicate an increased probability of harm. RegulomeDB (16) assigns a classification score from 1a to 7, which reflects the regulatory impact of SNPs determined by eQTLs and chromatin markers. A score of 1a signifies the most compelling biological evidence for the SNP’s regulatory role. We additionally annotated whether each lead SNP fell within the extended major histocompatibility complex region (xMHC), which has been defined as chr6: 25–34 Mb, to facilitate immunogenetic interpretation (17). This study utilized the European ancestry reference cohort dataset from the third phase of the 1000 Genomes Project. The PLACO (v0.1.1 and v0.2.0) (18, 19) method was employed to identify potential pleiotropic SNVs for pairs of traits exhibiting significant genetic correlation. The absence of pleiotropy was evaluated using the intersection–union test with Bonferroni correction, yielding the final pleiotropic *P*-values. SNVs with *P*-values < 5 × 10^−8^ were considered significant pleiotropic variants. Following the PLACO analysis, the identified loci were examined in relation to nearby genes to explore the common biological mechanisms associated with this pleiotropic location. The shared biological pathways associated with these pleiotropic loci were explored by utilizing the PLACO findings and the genomic loci identified through MAGMA (20).

### 2.4 Genetic insights

SMR (21) and two-sample MR (22) were employed to pinpoint the genes linked to complex traits influenced by pleiotropy, leveraging the summary statistics from eQTL and pQTL datasets. SMR utilizes SNPs as instrumental variables to assess the causal impact of an exposure on an environmental outcome and to eliminate outcomes displaying heterogeneity. We conducted integrative causal inference analyses using two-sample MR and SMR. eQTL and pQTL instruments were obtained from eQTLGen and deCODE, respectively. Meanwhile, outcome GWAS summary statistics for iridocyclitis were acquired. For two-sample MR, genome-wide significant cis-QTL variants were selected as candidate instruments (*P* < 5 × 10−8) and pruned for independence via linkage disequilibrium (LD) clumping (r2 < 0.001, 10,000 kb) by using the 1000 Genomes Project (1000G) reference panel. The number of retained instruments per exposure was reported. Primary MR estimates were obtained through inverse-variance weighted (IVW) analysis, and robustness was assessed by employing Cochran’s Q heterogeneity test and the MR–Egger intercept test for directional horizontal pleiotropy, with *P* > 0.05 indicating no evidence of substantial heterogeneity or directional pleiotropy. For SMR, we integrated the outcome GWAS with eQTLGen and deCODE QTL summary data by using 1000G for LD estimation, applying standard QC, including MAF ≥ 0.01 and allele-frequency consistency control (diff-freq-prop = 0.9). SMR effect estimates were reported together with heterogeneity in dependent instruments (HEIDI) heterogeneity results and the number of SNPs used in HEIDI as a colocalization/robustness metric. *P* < 0.05 was utilized as an exploratory threshold, and full MR/SMR results with robustness metrics were provided for transparency. Phenotypic and genomic enrichment analyses were conducted to evaluate the biological relevance of the genes linked to iridocyclitis. MAGMA was utilized for phenotypic enrichment analysis (17, 18). Genomic enrichment analyses were based on the data sourced from the Kyoto Encyclopedia of Genes and Genomes (KEGG) pathway and Gene Ontology (GO) pathway databases (22, 23).

### 2.5 Proteomic insights

To unravel the complex proteomic profiles linked with iridocyclitis and immune-mediated diseases, this study utilized the BLISS technique (6). BLISS is a novel approach for developing protein interpolation models directly from summary-level pQTL data, thereby enabling the creation of comprehensive European proteome-wide association study (PWAS) models by employing extensive pQTL information from large repositories, including the UKB and ARIC studies. In this research segment, proteins exhibiting an FDR of less than 0.05 were recognized as significant risk proteins, highlighting their potential role in the pathophysiology of iridocyclitis and related immune-mediated diseases. Enrichment analysis and protein–protein interaction network (PPI) analysis were performed using Cytoscape and String platform (https://string-db.org/).

### 2.6 Construction of drug–gene interaction networks

The Drug–Gene Interaction Database (DGIdb, http://www.dgidb.org/, accessed on May 28, 2024) (24) was utilized to predict interactions between FDA-approved drugs and pleiotropic genes and conduct drug enrichment analyses using intersection outcomes.

### 2.7 Gene set variation analysis, machine learning, and immune cell infiltration analysis

Gene expression data were extracted from four datasets (GSE73754, GSE94648, GSE209567 and GSE134025) from the Gene Expression Omnibus (GEO) database (25). Three machine learning techniques (random forest, support vector machine, and LASSO regression) were employed to identify pleiotropic genes. Immune cell infiltration analysis and GSVA (26) were then conducted using the gene screening outcomes obtained from the three methods.

### 2.8 SceQTL analysis

Seventeen single-cell eQTLs (sceQTLs) of immune cells were extracted from the scQTLbase (http://bioinfo.szbl.ac.cn/scQTLbase/Home/). Linkage disequilibrium (KB = 100, r^2^ = 0.1, *P*1 < 0.05, *P*2 = 1) was eliminated for two-sample MR analysis.

### 2.9 Flow cytometer

Murine RAW264.7 macrophages (CL-0190) were cultured in Dulbecco’s Modified Eagle Medium (DMEM, Gibco) supplemented with 10% fetal bovine serum (FBS, Gibco) and 1% penicillin-streptomycin (Gibco) at 37°C in a 5% CO_2_ atmosphere. Cells were seeded in 24-well plates at a density of 1×10^5^ cells per well. After overnight adhesion, cells were stimulated with 1 μg/mL lipopolysaccharide (LPS, Sigma-Aldrich, L2630) for 24 hours; unstimulated cells served as controls.

For flow cytometry analysis, separate sets of identically treated cells were used for the detection of IL15RA and FBXL18. All cells were harvested and washed twice with cold PBS. To detect surface IL15RA, cells were incubated with an unlabeled rabbit anti-mouse IL15RA antibody (Invitrogen, PA5-79467, 1:200) for 30 minutes at 4°C, followed by an Alexa Fluor 488-conjugated goat anti-rabbit IgG secondary antibody (Invitrogen, A-11008, 1:400) for 30 minutes at 4°C. To detect intracellular FBXL18, a separate set of cells was first fixed and permeabilized by incubation with the Fixation/Permeabilization Concentrate (Invitrogen, 00-5123-43, diluted 1:3) for 60 minutes at 4°C. After washing, cells were then incubated with a rabbit anti-FBXL18 antibody (Zeye Biotechnology, ZY-6802-89R, 1:200) for 60 minutes at 4°C, followed by the same AF488-conjugated secondary antibody (1:400) for 30 minutes at 4°C. For negative controls, cells were processed identically but incubated with the secondary antibody alone (secondary antibody control). The negative population for gating was defined based on this control. All steps were performed in the dark, with washes between incubations.

Following final washes, cells were resuspended in 300 μL of PBS and analyzed on a BD Accuri C6 flow cytometer. A minimum of 10,000 events per sample were acquired. Data analysis was performed using FlowJo software (v10.8.1). The intact cell population was initially gated on a forward scatter-area (FSC-A) versus side scatter-area (SSC-A) plot to exclude debris. Single cells were then selected by gating on FSC-A versus forward scatter-height (FSC-H) to exclude doublets. The mean fluorescence intensity (MFI) of the target proteins within this singlet gate was used for quantification.

Data are presented as mean ± SEM from four independent experiments. Statistical significance was determined using an unpaired Student’s t-test, with a *P*-value < 0.05 considered statistically significant.

### 2.10 Software information

The information of the analytical tools used in this study is as follows: R (v4.4.2; Ubuntu 24.04.2 LTS), METAL (MetaAnalysis Helper; version 2011-03-25), FUMAGWAS (v1.8.0), MAGMA (v1.10), SMR (v1.3.1), TwoSampleMR (v0.6.8), PLACO (v0.1.1 and v0.2.0) and BLISS (v1.0.2). The reference panel used in the study was the 1000 Genomes Project (1000G) EUR population data.

## 3 Results

### 3.1 GWAS-meta

**Figure 2:**
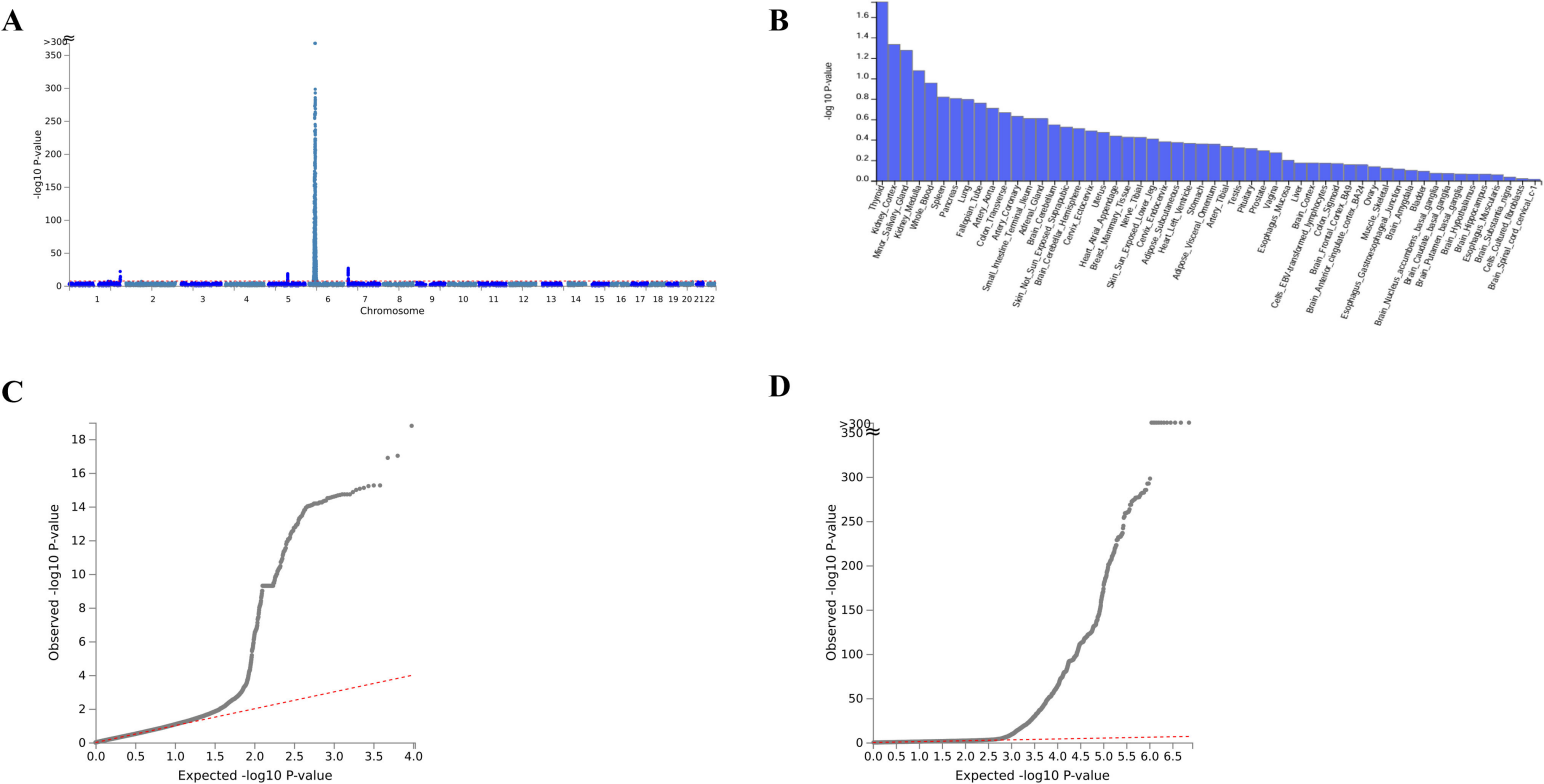
Functional mapping and annotation of meta-iridocyclitis. **(A)** Manhattan plots:The x-axis shows chromosomal position, and the y-axis shows association p-values on a −log10 scale. **(B)** MAGMA Tissue Expression Analysis (GTEx v8–53 tissue types): The x-axis shows 53 tissue, and the y-axis shows association p-values on a −log10 scale, Whole blood shows the most significant enrichment. **(C)** QQ plot (GWAS summary statistics). **(D)** QQ plot (gene-based).

**Figure 3:**
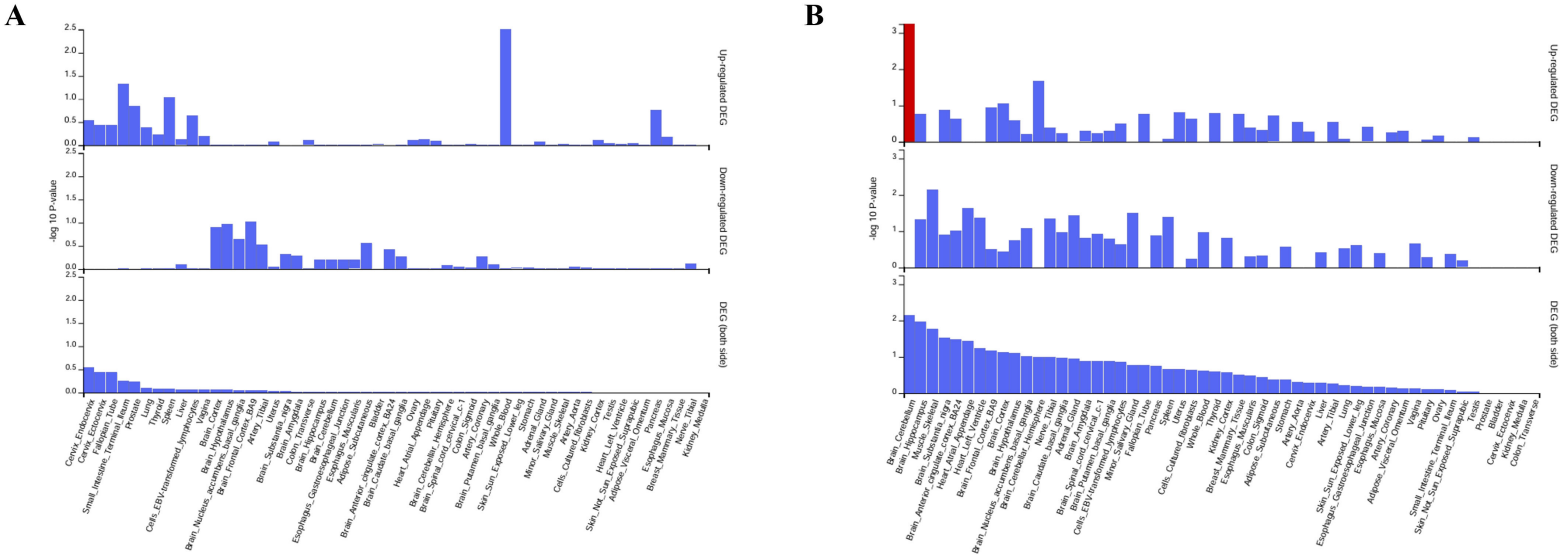
Meta-iridocyclitis mapped genes and multiple disease risk genes. **(A)** Tissue Expression Analysis (GTEx v8–54 tissue types): The x-axis shows 53 tissues, and the y-axis shows association p-values on a −log10 scale. **(B)** Tissue Expression Analysis of Multiple disease risk genes: The x-axis shows 53 tissues, and the y-axis shows association p-values on a −log10 scale.

GWAS meta-analysis was conducted using data from the FinnGen database (R11), the IEU database, and the GWAS catalog database, focusing on four diseases: iridocyclitis, ulcerative colitis, Crohn’s disease, and ankylosing spondylitis. The aggregated data resulted in four GWAS summary statistics (META-iridocyclitis, META-ulcerative colitis, META-Crohn’s disease, and META-ankylosing spondylitis) for subsequent PLACO analysis (**Supplementary Table S1**). The PLACO analysis identified 118 significant SNPs associated with iridocyclitis-ulcerative colitis, 101 significant SNPs associated with iridocyclitis-Crohn’s disease, and 805 significant SNPs associated with iridocyclitis-ankylosing spondylitis (**Supplementary Table S2**). FUMA identified five genomic risk loci for META-iridocyclitis (**Table 2**), with the lead variants rs145759877 (1:248246983), rs142283392 (5:96101518), rs190162014 (6:29249738), rs181454372 (6:33309110), and rs140643804 (7:5566486). Two loci (rs190162014 and rs181454372) were mapped to the xMHC region (chr6: 25–34 Mb), consistent with the highly polymorphic and LD-dense HLA region. Given the extensive LD and gene density in this region, causal gene attribution should be interpreted cautiously. The remaining loci are outside xMHC; notably, the locus tagged by rs142283392 is proximal to the ERAP1/CAST region, which has been repeatedly implicated in immune-mediated inflammatory diseases (IMIDs), supporting potentially shared immunogenetic pathways. Additionally, FUMA analysis mapped 123 genes on the basis of specific screening criteria (**Supplementary Table S3**; **Table 2**) (**Figures 2**, **3**; **Supplementary Figure 1**).

**Table 2:** Genomic risk loci identified for meta-iridocyclitis by FUMA and their overlap with the extended MHC region.

SNP	Chr: Pos	Locus start–end	MHC
rs145759877	1:248246983	247687927–249215865	No
rs142283392	5:96101518	96033870–96223552	No
rs190162014	6:29249738	22432653–29614419	Yes
rs181454372	6:33309110	33173842–39300928	Yes
rs140643804	7:5566486	4434488–6899213	No

Chr: Pos, Chromosome:base-pair Position; MHC, Major Histocompatibility Complex; FUMA, Functional Mapping and Annotation.

Quality control using LDSC indicated mild genomic inflation for META-iridocyclitis (mean χ2 = 1.0276; λGC = 1.0224) with an LDSC intercept of 1.0107 (SE = 0.0069), suggesting limited residual confounding. Bivariate LDSC cross-trait intercepts between META-iridocyclitis and each immune-mediated disease were 0.0251 (SE = 0.0050) for Crohn’s disease, 0.0154 (SE = 0.0046) for ulcerative colitis, and 0.1095 (SE = 0.0053) for ankylosing spondylitis (**Table 3**). We employed these estimates to quantify potential sample overlap confounding and guide the conservative interpretation of cross-trait analyses.

**Table 3:** Cross-trait linkage disequilibrium score regression intercepts (standard errors) to assess potential sample overlaps between Iridocyclitis and three immune-related diseases.

Trait 1	Trait 2	Cross-trait intercept (SE)
Iridocyclitis	Crohn	0.0251 (0.0050)
	Ulcerative colitis	0.0154 (0.0046)
	Ankylosing spondylitis	0.1095 (0.0053)

SE, Standard Errors.

### 3.2 Gene enrichment analysis and tissue enrichment analysis

**Figure 4:**
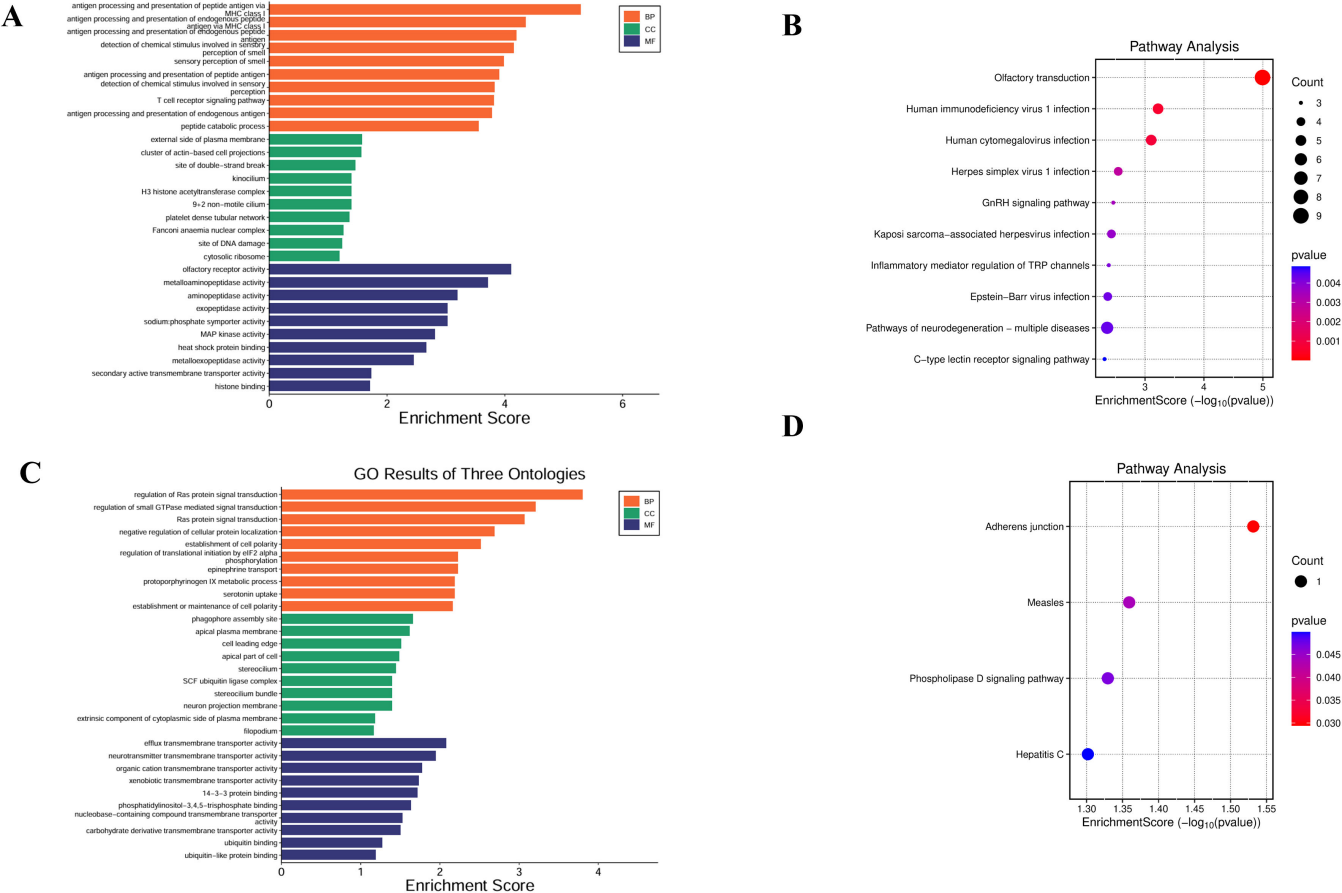
Enrichment analysis for the meta-iridocyclitis mapped genes and multiple disease risk genes. **(A, C)** GO: BP, biological process; CC, cellular component; MF, molecular function; **(B, D)** KEGG: KEGG, Kyoto encyclopedia of genes and genomes pathway.

**Figure 5:**
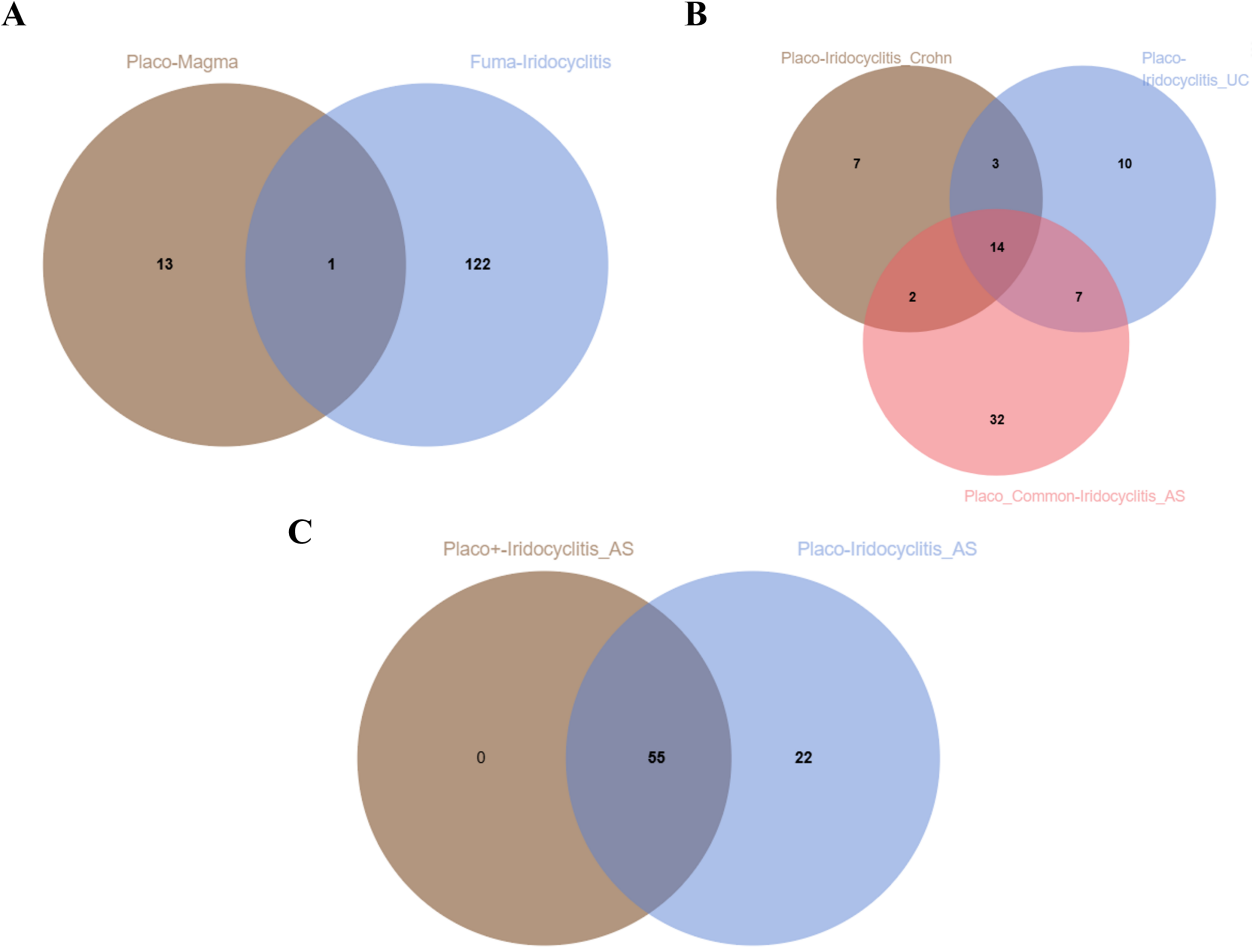
Shared gene venn map. **(A)** Meta-Iridocyclitis mapped genes and Multiple disease risk genes. **(B)** PLACO analysis for the Meta-Iridocyclitis and Meta-Crohn, Meta-Iridocyclitis and Meta-Ulcerative Colitis, Meta-Iridocyclitis and Meta-Ankylosing Spondylitis. **(C)** PLACO analysis for the Meta-Iridocyclitis and Meta-Ankylosing Spondylitis VS PLACO+ analysis for the Meta-Iridocyclitis and Meta-Ankylosing Spondylitis.

On the basis of the molecular QTL summary data from eQTLGen (eQTL) and deCODE (pQTL), we conducted molecular-level causal inference for META-iridocyclitis by using SMR and two-sample MR. A total of 260 eQTL genes and 48 pQTL were identified to be nominally associated with META-iridocyclitis(*P* < 0.05). We reported the number of instrumental variables, heterogeneity tests, and horizontal pleiotropy assessment for each exposure in the MR results to assess the robustness of the results. In the nominally significant candidate associations, the *P* values for heterogeneity and pleiotropy tests were all >0.05, and no obvious evidence of heterogeneity or directional horizontal pleiotropy was observed. For SMR, we also reported *P*_HEIDI and nsnp_HEIDI as robustness indicators to assist in distinguishing potential colocalization signals from LD-driven associations (**Supplementary Table S4**). Enrichment analysis of the META-iridocyclitis mapping gene revealed significant enrichment in biological processes including antigen processing and peptide antigen presentation by MHC class I molecules. Tissue enrichment analysis indicated enrichment across various tissues, with whole blood exhibiting the most pronounced enrichment. Similarly, enrichment analysis of multiple disease risk genes demonstrated significant enrichment in regulation of ras protein signal transduction (**Figure 4**). Tissue enrichment analysis revealed enrichment across multiple tissues, with Brain_Cerebellum enrichment being the most notable (**Figure 3**). To minimize potential bias in the bivariate analysis arising from sample overlap between META-iridocyclitis and AS, we performed a cross-trait co-localization analysis using PLACO+ (v0.2.0). PLACO+ ensures robust statistical inference by explicitly modeling the variance and correlation structure of Z-scores for the two traits under the null hypothesis, thereby accounting for sample overlap or correlated errors. The correlation structure was estimated using weakly associated SNPs, with a significance threshold of *P*_est_thr = 1×10e−4. Based on this approach, we obtained estimates of trait-specific Z-score variances (VarZ_IR = 1.0036; VarZ_TR2 = 0.9785) and their inter-trait correlation coefficient (CorZ = 0.1388). To mitigate the confounding effects of high-linkage disequilibrium immune-related regions, the MHC region (chr6: 25–34 Mb) was excluded from the analysis. Furthermore, to reduce ambiguity in allele orientation, palindromic SNPs (A/T or C/G) were removed; additionally, palindromic variants with minor allele frequency (MAF) near 0.5 were excluded using a threshold of *P*al_maf_low = 0.42. During correlation parameter estimation, extreme test statistics were filtered out by excluding SNPs with Z² > 80 (z2_thr = 80) to prevent inflation due to strongly associated loci. Finally, we intersected the results from PLACO and PLACO+ analyses (Placo-iridocyclitis_AS and Placo+-iridocyclitis_AS) (**Figure 5C**, **Supplementary Table S5**). Disease-associated SNPs identified through PLACO analysis were further annotated using MAGMA analysis, revealing 34 genes significantly linked to iridocyclitis-ulcerative colitis, 26 genes to iridocyclitis-Crohn’s disease, and 55 genes to iridocyclitis-ankylosing spondylitis. Through the intersection of related genes post-PLACO analysis, a total of 14 multi-disease risk genes were pinpointed (**Supplementary Table S5**, **Figure 5B**). Notably, the iridocyclitis mapping gene overlaps with one gene (FBXL18) found in multiple disease risk genes (**Figure 5A**).

### 3.3 Construction of drug–gene interaction networks

**Figure 6:**
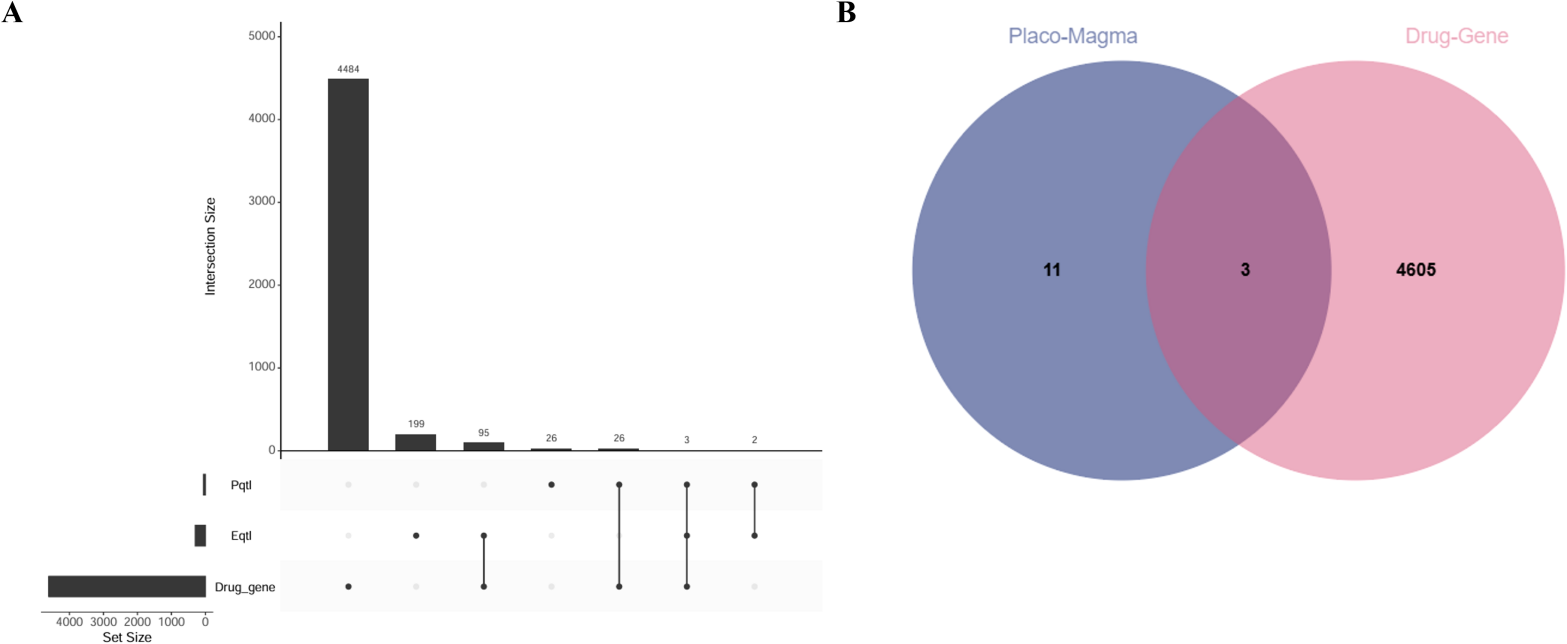
Shared gene venn and upset map. **(A)** Meta-Iridocyclitis related eQTL, pQTL and druggable genes. **(B)** Multiple disease risk genes and druggable genes.UC, Ulcerative Colitis, AS, Ankylosing Spondylitis.

**Figure 7:**
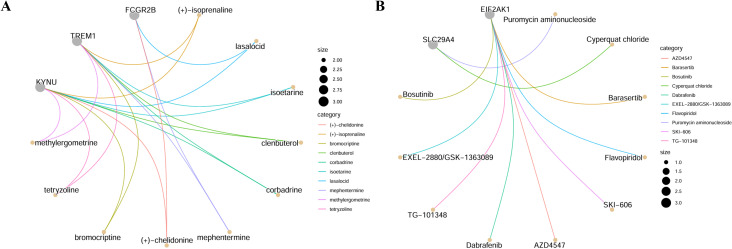
The drug–gene interaction network of the annotated genes through DGIdb. **(A)** Intersection genes of the Meta-Iridocyclitis related eQTL, pQTL and druggable genes **(B)** Intersection genes of Multiple disease risk genes and druggable genes.

Drug enrichment analysis was conducted on three iridocyclitis-related drug-genes intersection genes (FCGR2B, KYNU, TREM1) derived from the overlap of iridocyclitis-related eQTL, pQTL, and drug-genes. The analysis revealed interactions between 70 drugs and the three intersection genes (**Figures 6**, **7**, **Supplementary Document S4**). Similarly, drug enrichment analysis was carried out on drug-susceptible intersection genes (EIF2AK1, RIPOR2, SLC29A4) identified from the intersection of multiple disease risk genes and drug-susceptible genes, demonstrating interactions between 12 drugs and the three intersection genes (**Figures 6**, **7**, **Supplementary Table S6**).

### 3.4 GSVA, machine learning and immune cell infiltration analysis

**Figure 8:**
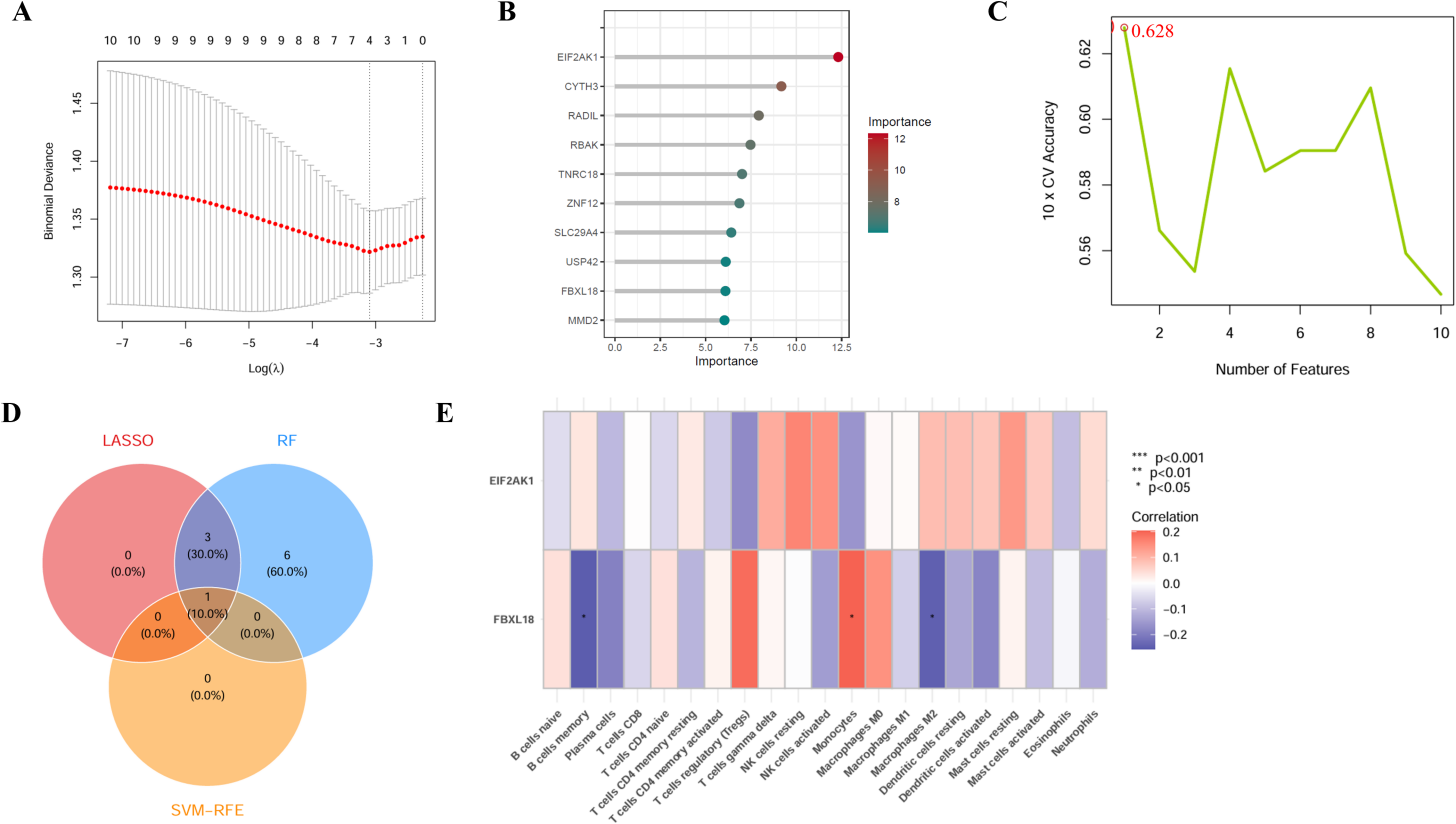
Machine learning analysis, immune cell infiltration and sceQTL. **(A)** LASSO regression: The x-axis shows log(λ), and the y-axis shows binomial deviance. **(B)** Random Forest: The x-axis shows gene score in the model, and the y-axis shows gene. **(C)** Support Vector Machine: The x-axis shows number of genes in the model, and the y-axis shows cross-validation accuracy. **(D)** Three Machine Learning Intersection Genes: RF, Random Forest, SVM, Support Vector Machine. **(E)** Immune cell infiltration analysis: The x-axis shows Immune cell, and the y-axis shows gene.

**Figure 9:**
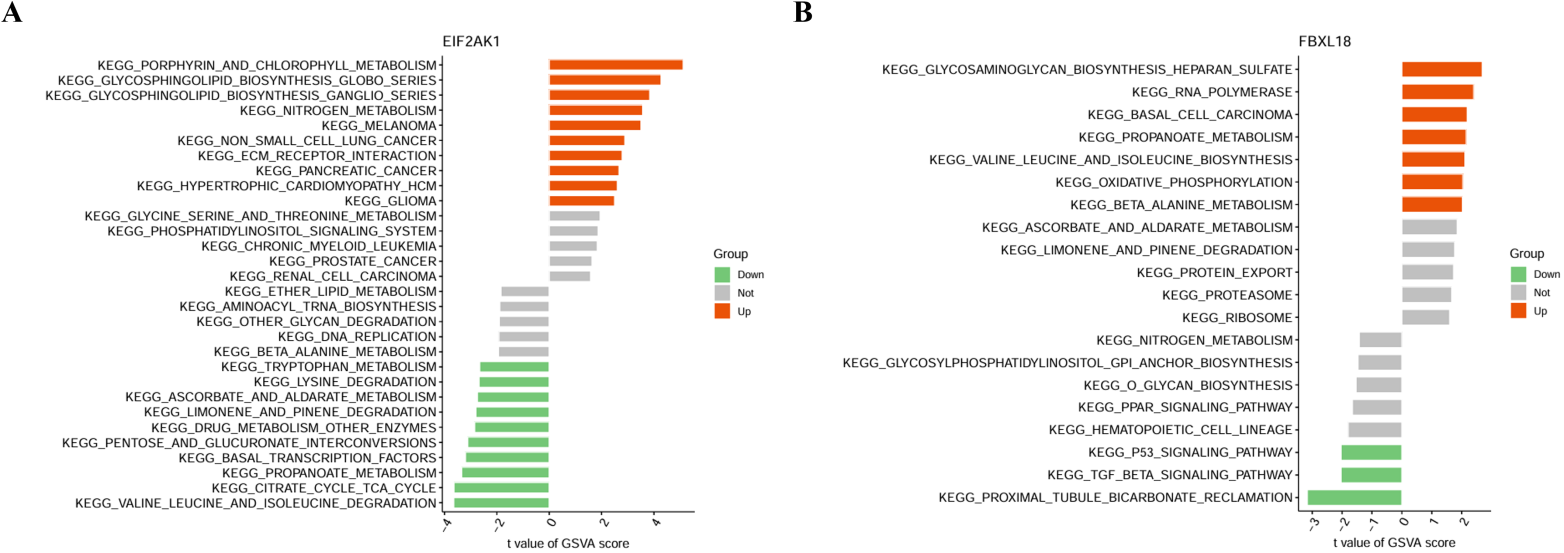
Plot of gene set variation analysis. **(A)** EIF2AK1, **(B)** FBXL18.

The study extracted multi-disease risk gene expression results from a combined dataset comprising four datasets (GSE73754, GSE94648, GSE209567, and GSE134025) in the GEO database, yielding data on 10 genes. Through the application of random forest, SVM, and Lasso algorithms EIF2AK1 was identified. Subsequent immunocyte infiltration analysis indicated FBXL18 is significantly expressed in monocytes and exhibits an increasing trend in regulatory T cells (Tregs). GSVA analysis revealed that FBXL18 exhibits a significant upregulation in key biological pathways, including glycosaminoglycan biosynthesis, RNA polymerase activity, propionate metabolism, and amino acid metabolism., EIF2AK1 in porphyrin and chlorophyll metabolism (**Figures 8**, **9**, **Supplementary Table S7**).

### 3.5 SceQTL analysis

**Figure 10:**
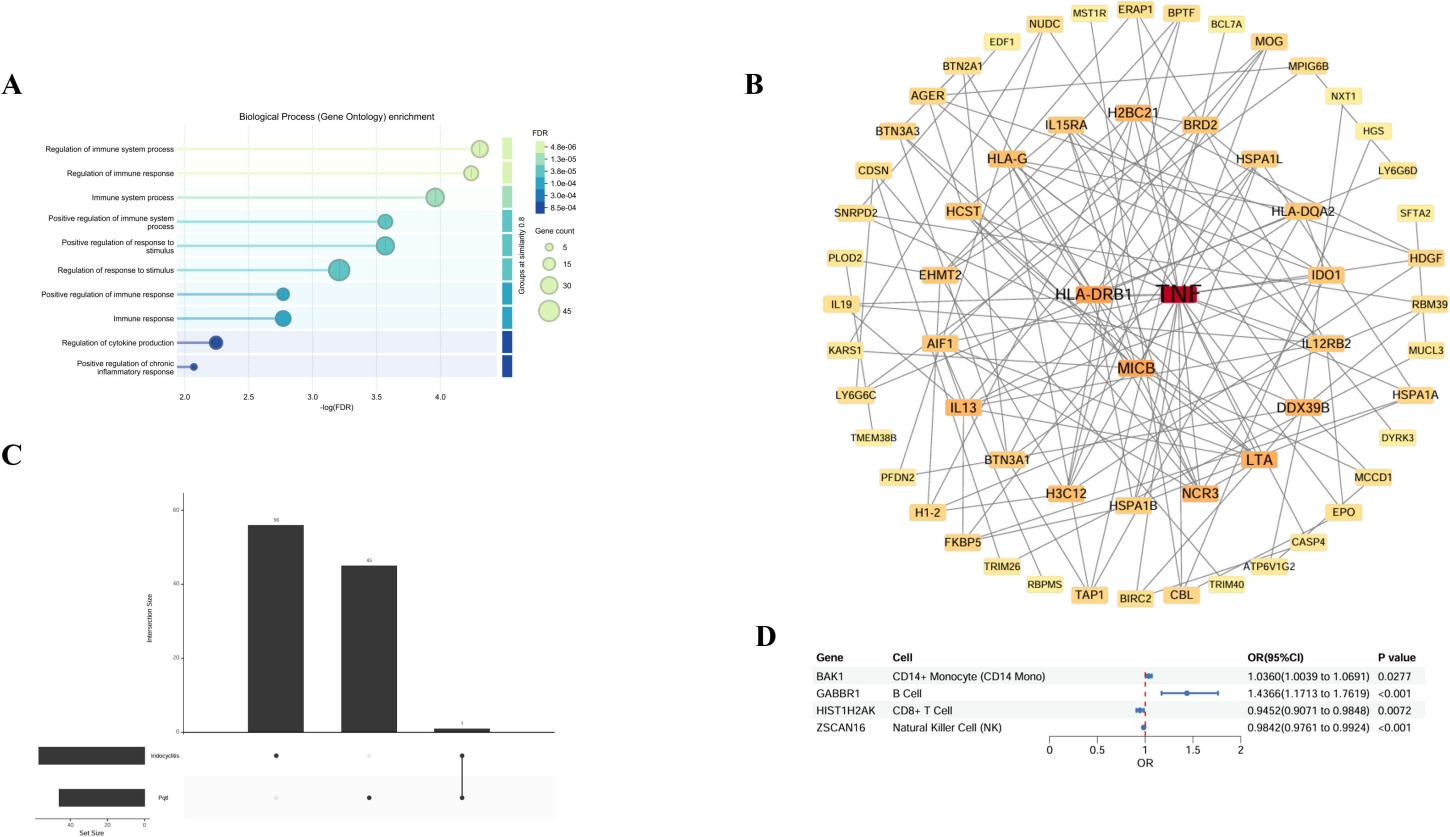
Plot of disease-related proteins **(A)** Protein enrichment analysis; **(B)** PPI network diagram; **(C)** Plot of Upset. **(D)** sceQTL forest plot: sceQTL, Single Cell eQTL. BLISS, Biomarker Imputation from Summary Statistics; MR, Mendelian Randomization; SMR, Summary-based Mendelian randomization.

We extracted SceQTL data from immune cells using genes mapped to iridocyclitis, resulting in 26 gene-cell associations. Two-sample Mendelian randomization was employed to investigate the connection between immune cell eQTL and iridocyclitis. Our analysis revealed significant associations between 4 genes, immune cells, and iridocyclitis. (BAK1-CD14+ Monocyte (CD14 Mono), GABBR1-B Cell, HIST1H2AK-CD8+, ZSCAN16-Natural Killer Cell (NK)) (**Figure 10D**, **Supplementary Table S8**).

### 3.6 Protein enrichment analysis and PPI

Based on the BLISS method combined with Meta data, we evaluated 2802, 4418 plasma proteins in the UKB and ARIC, respectively. The results revealed a total of 46 proteins were associated with iridocyclitis, 52 with AS, 18 with Crohn’s disease, and 15 with UC. Enrichment analysis of these proteins revealed significant enrichment in the Regulation of Immune System process, with TNF playing an important role in the PPI. In addition, the BLISS analysis results, MR, and SMR results together demonstrated the close relationship between IL15RA and iridocyclitis (**Supplementary Table S9**, **Figure 10**).

### 3.7 Flow cytometry validation of FBXL18 and IL15RA expression

**Figure 11:**
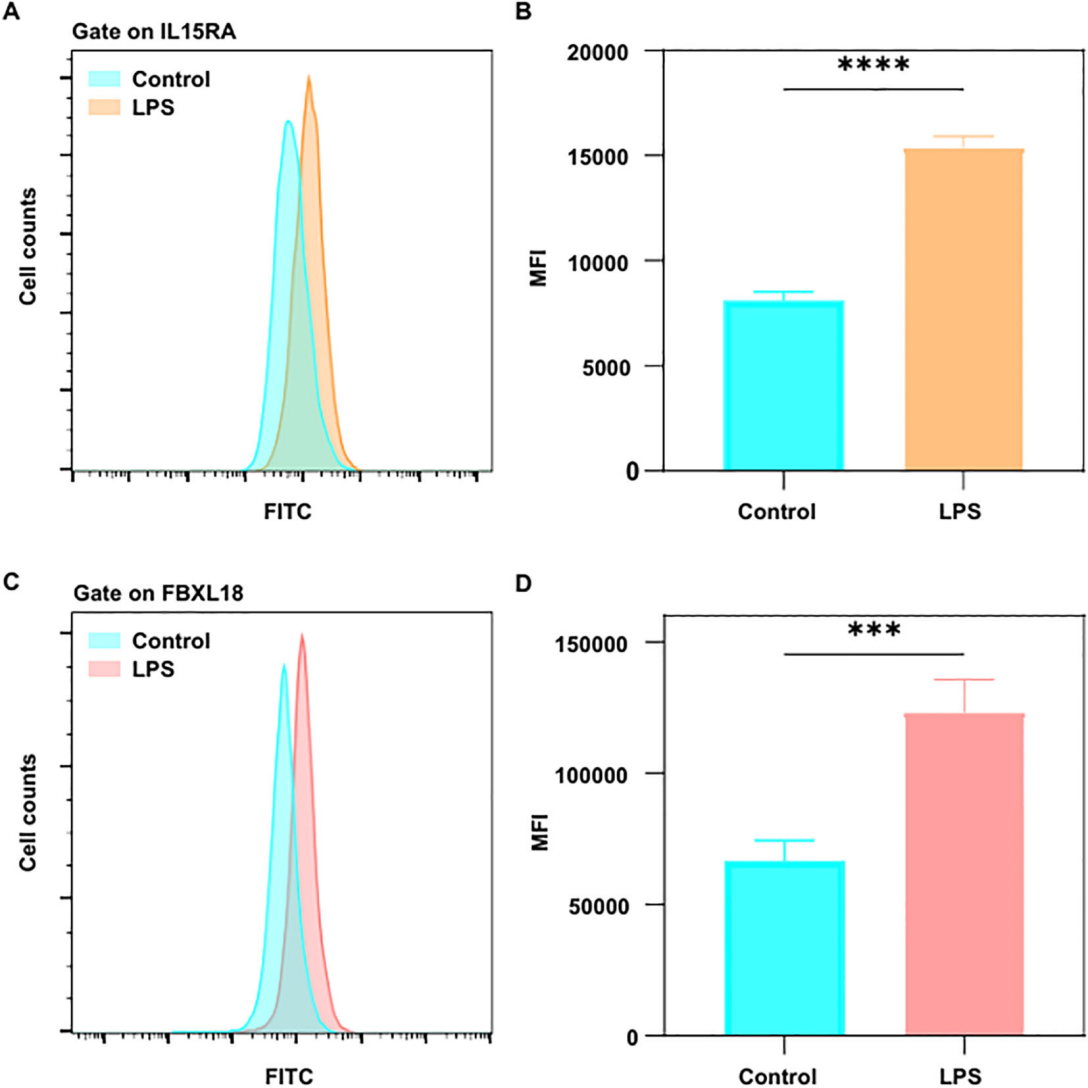
LPS upregulates the expression of IL15RA and FBXL18 in RAW 264.7 macrophages. **(A, C)** Representative flow cytometry histograms showing the expression levels of **(A)** IL15RA and **(C)** FBXL18 in unstimulated control cells (blue) and cells stimulated with LPS (1 μg/mL, orange/pink) for 24 hours. Fluorescence was detected using the FITC channel. **(B, D)** Quantitative analysis of the mean fluorescence intensity (MFI) for **(B)** IL15RA and **(D)** FBXL18. Data are presented as mean ± SEM (n = 4 independent experiments). Statistical significance was determined by Student’s t-test. ***P < 0.001, ****P < 0.0001.

To further validate the genetic findings, we performed flow cytometry to assess the protein expression of FBXL18 and IL15RA in Raw 264.7 cells following LPS stimulation. LPS treatment for 24 hours significantly increased the expression levels of both FBXL18 and IL15RA, as indicated by elevated MFI compared to unstimulated controls (**Figure 11**). This result confirms that FBXL18 and IL15RA are responsive to inflammatory stimuli and supports their role in immune-mediated pathways relevant to iridocyclitis.

## 4 Discussion

Patients with iridocyclitis often present with cooccurring immune-mediated diseases that share genetic and immunological similarities mainly driven by helper T-cell response (27). A common feature of these diseases is their immune pathogenesis, characterized by hyperactivity within specific components of the immune system (28, 29). This shared, complex genetic architecture necessitates an integrated analytical approach, rather than a traditional single-disease framework, to identify the genetic factors influencing both conditions. This study employed an innovative, comprehensive method to investigate the genetic relationship between iridocyclitis and four related diseases.

Iridocyclitis is not a single uniform entity but an umbrella phenotype encompassing multiple clinical subtypes with different triggers, disease courses, and systemic associations. Therefore, genetic analyses based on a broad iridocyclitis definition may preferentially capture loci shared across inflammatory pathways, whereas subtype-specific signals could be diluted. Accordingly, shared loci identified with AS, CD, and UC should be interpreted as indicating overlapping immunogenetic mechanisms and/or overlapping systemic disease–associated subgroups rather than complete etiologic equivalence across all forms of iridocyclitis.

GWAS data from various sources were pooled to examine four diseases, with prior research indicating a strong link among iridocyclitis, UC, CD, and AS. By utilizing PLACO, MAGMA, gene enrichment, tissue enrichment, ML, and immune cell infiltration analyses, this study investigated the characteristics of multidisease risk genes. The gene EIF2AK1 was identified. EIF2AK1 is primarily recognized for linking heme availability with globin synthesis in early erythroid cells (30). Beyond erythroid tissues, EIF2AK1 modulation induces physiological changes in other tissues. EIF2AK1 knockout elevates ER stress in mouse hepatocytes (31), whereas its pharmacological activation mitigates ER stress-induced hepatic steatosis and glucose intolerance in mouse models (32). Endoplasmic reticulum stress is strongly associated with a variety of inflammatory diseases (33–36). This study identified a significant increase in EIF2AK1 expression within porphyrin and chlorophyll metabolism. Porphyrins, known as endogenous anti-inflammatory agents, exhibit synthetic, analgesic, and anti-inflammatory properties (37). Chlorophyll and its derivatives mitigate adenine-induced chronic kidney disease by inhibiting TGF-β and inflammatory cytokines (38).

Although the relationship between the three multidisease risk genes and the four inflammatory diseases examined in this study has not been precisely delineated, our findings and those from earlier works suggest these genes’ potential role in inflammatory conditions. Gene screening and mapping focused on meta-iridocyclitis identified 123 genes notably enriched in biological processes, including antigen processing and peptide antigen presentation via MHC class I molecules. Significant tissue enrichment was also observed, particularly in whole blood. By intersecting the meta-iridocyclitis mapping genes with the multidisease risk genes, we identified the consensus gene FBXL18. FBXL18 facilitates the ubiquitination and proteasome degradation of proteins, such as targeting LRRK2 to mitigate cytotoxicity (39), mediating polyubiquitination and proteasome degradation of the pro-apoptotic SCF subunit Fbxl7 (40) and promoting RPS15A ubiquitination (41). Ubiquitination is also closely related to ocular inflammation (42). This finding demonstrates the potential of the gene in inflammatory diseases. In addition, this study found that FBXL18 is significantly upregulated in monocytes, a finding consistent with a recent report in Nature Communications. The study demonstrated that in rabies virus-infected astrocytes, FBXL18 is markedly induced and stabilizes BST2 through K11-linked ubiquitination, leading to excessive phosphorylation of IκBα and consequent hyperactivation of NF-κB, which promotes the production of proinflammatory mediators such as Interleukin-6 (IL-6) and Interferon gamma-induced protein 10 (CXCL10). Furthermore, knockdown of FBXL18 effectively suppresses IL-6 expression and attenuates the inflammatory phenotype. These findings collectively underscore the critical role of FBXL18 in inflammatory processes and suggest its potential as a therapeutic target for iridocyclitis (43).

sceQTL analysis was conducted to investigate the genetic mechanisms underlying meta-iridocyclitis. The following four genes and their associated immune cells significantly linked to iridocyclitis were identified: BAK1 with CD14+ monocytes, GABBR1 with B cells, HIST1H2AK with CD8+ T cells, and ZSCAN16 with natural killer (NK) cells. CD14+ monocytes are implicated in inflammatory responses across various diseases. For instance, in psoriasis, miR-155 inhibition reduces CD14+ monocyte-mediated inflammation and oxidative stress (44). In NSCLC, the interleukin-33/ST2 axis enhances lung-resident CD14+ monocyte function (45). B cells and CD8+ T cells are crucial in rheumatic diseases; G protein-coupled receptor 40 is pivotal in B cell responses in mice and humans, thus influencing rheumatoid arthritis pathogenesis (46). In Wu et al.’s study (47), single-cell nuclear transcriptomics revealed a strong extrafollicular B cell response in lupus kidneys linked to granzyme K CD8 T cell activation. NK cells are closely associated with tumors and the tumor immune microenvironment. Fernando et al. (48) found that TGF-β and CIS inhibition can override NK cell suppression, restoring antitumor immunity.

Iridocyclitis arises from the abnormal activation of Th1 cells, which primarily secret IFN-γ, and Th17 cells, which primarily secrete IL-17. These cells breach the blood–retinal barrier, recruiting inflammatory cells such as macrophages and monocytes, leading to ocular inflammation and tissue damage. B lymphocytes also contribute to iridocyclitis; for instance, STAT3 deficiency in B cells exacerbates the condition by promoting pathogenic lymphocyte expansion and inhibiting regulatory B cells and T cells (49). These findings underscore the complex biological mechanisms of iridocyclitis and highlight the roles of T and B lymphocytes. This study also identified several inflammation-related genes not previously associated with iridocyclitis, offering new insights into its pathogenesis. Analysis using the FUMA platform further identified five independent risk loci for iridocyclitis (rs145759877, rs142283392, rs190162014, rs181454372, and rs140643804). These loci, not extensively detailed in prior studies, may serve as potential targets for future iridocyclitis treatments. Notably, among the five independent risk loci identified by FUMA, rs190162014 (chr6: 29.25 Mb) and rs181454372 (chr6: 33.31 Mb) are located in the xMHC (6p21) region, which is consistent with the strongest association peak shown in **Figure 2A**. As a result of the high polymorphism and strong LD structure of the MHC region, the lead SNP in GWAS often reflects the tagging effect of HLA haplotypes/alleles, complicating locating specific HLA alleles precisely on the basis of SNPs alone. In terms of genomic location, the 29–31 Mb range is close to the HLA class I–adjacent segment, whereas the 33–34 Mb range is close to the HLA class II–adjacent segment. This situation suggests that genetic susceptibility to META-iridocyclitis may involve class I and II antigen presentation pathways. This finding is biologically consistent with the Th1/Th17-related immune activation emphasized in our study. This pattern also contrasts with previous evidence: iridocyclitis and AS often shows class I (such as HLA-B27) dominance, whereas the MHC signals in IBD are inclined toward class II. In addition, we detected a significant locus containing ERAP1 (rs142283392) in the non-MHC region, further supporting the potential role of the antigen processing–presentation axis in disease occurrence. Given that we were limited by the data type, we are still unable to attribute the xMHC signals to specific HLA alleles. Subsequent analysis through HLA inference, MHC conditional analysis, and fine mapping can further clarify the independent contributions and interrelationships of classes I and II (50–52). To investigate gene–drug interactions, we identified the eQTLs and pQTLs associated with iridocyclitis using MR and SMR analyses, revealing three intersecting genes: FCGR2B, KYNU, and TREM1. Drug enrichment analysis highlighted five closely related drugs: (+)-isoprenaline, lasalocid, isoetarine, clenbuterol, and corbadrine. Further analysis of disease risk genes and drug-ready genes identified three additional intersection genes: EIF2AK1, RIPOR2, and SLC29A4. Drug enrichment linked these genes to four drugs: puromycin aminonide, cypriquat chloride, barasertib, and flavopiridol, suggesting potential new treatment avenues for inflammatory diseases. Using the BLISS method combined with Meta data, we evaluated 2802, 4418 plasma proteins in UKB and ARIC, respectively. The results revealed 46 proteins associated with iridocyclitis, 52 with AS, 18 with CD, and 15 with UC. Enrichment analysis of these proteins revealed significant enrichment in the regulation of immune system processes, with TNF playing an important role in the PPI. The results of BLISS, MR, and SMR demonstrated the close relationship between IL15RA and iridocyclitis. Zhu et al. (53) found that blocking IL-15 can specifically deplete the memory CD4+ T cells generated by uveitis and break the chronic disease state of uveitis, which is of great value for the treatment of this disease. This finding demonstrates the importance of the IL-15/IL15RA pathway. From a therapeutic perspective, IL-15 antagonists may be included in the treatment of uveitis in the future.

This study underscores the significance of multifunctional genes in identifying iridocyclitis and elucidating the roles of various immune cells in its pathogenesis. The identified pleiotropic sites identified offer promising targets for developing precise iridocyclitis therapies. Our study has limitations. First, iridocyclitis is a clinically heterogeneous phenotype spanning distinct etiologic and immunopathologic subtypes. Such phenotype heterogeneity may attenuate effect estimates; dilute subtype-specific genetic signals; and complicate the interpretation of shared loci with AS, CD and UC, which may be driven by overlapping systemic disease–associated subgroups (HLA-B27-related disease). Second, the GWAS dataset was sourced from individuals of European descent, limiting its generalizability. Therefore, multiethnic studies are warranted. Although the genes linked to iridocyclitis and immune-mediated disease comorbidities were identified, the potential influence of epigenetic modifications on gene expression and disease phenotypes remain unexplored. Future longitudinal and experimental studies are crucial to understanding these biological mechanisms fully.

## 5 Conclusion

Ultimately, this research reveals a close genetic relationship between iridocyclitis and three immune-mediated diseases, identifying novel genetic risk factors and enhancing the understanding of iridocyclitis’s genetic landscape, which could aid in devising innovative treatments.

## Data Availability

The datasets presented in this study can be found in online repositories. The names of the repository/repositories and accession number(s) can be found in the article/**Supplementary Material**.

## Supplementary material

Supplementary Figure 1 Heat map: The x-axis shows the tissue, and the y-axis shows the gene.
