# Supplementary figures and images for: Identifying common genetic etiologies between iridocyclitis and related immune-mediated diseases
Source: Front Immunol. 2026 Jan 15;16:1755047. doi: 10.3389/fimmu.2025.1755047 (PMC12852376; doi:10.3389/fimmu.2025.1755047)

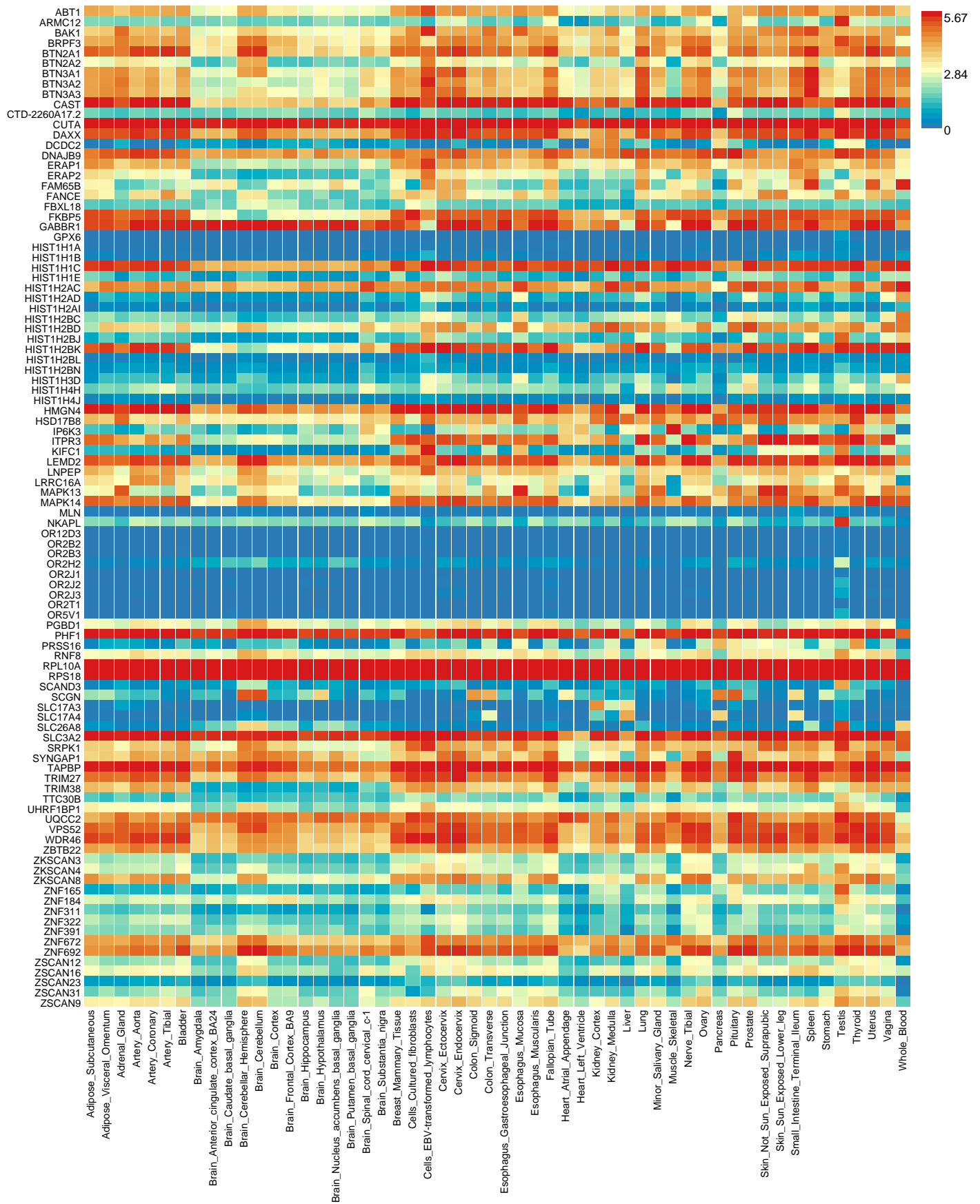

Supplement: Supplementary Figure 1 — Heat map: The x-axis shows the tissue, and the y-axis shows the gene. [file DataSheet1.pdf]
